# Correlation between Thermal Behaviour of AA5754-H111 during Fatigue Loading and Fatigue Strength at Fixed Number of Cycles

**DOI:** 10.3390/ma11050719

**Published:** 2018-05-02

**Authors:** Rosa De Finis, Davide Palumbo, Livia Maria Serio, Luigi A. C. De Filippis, Umberto Galietti

**Affiliations:** Politecnico di Bari—Department of Mechanics Mathematics and Management (DMMM), 70126 Bari, Italy; davide.palumbo@poliba.it (D.P.); liviamaria.serio@poliba.it (L.M.S.); luigi.defilippis@poliba.it (L.A.C.D.F.); umberto.galietti@poliba.it (U.G.)

**Keywords:** fatigue, high diffusive materials, aluminium alloy 5754-H111, thermography, thermoelastic stress analysis

## Abstract

The characterization of the fatigue behaviour of aluminium alloys is still capturing the attention of researchers. As it is well known in literature, for certain alloys, in a specific range of cycles number, the S-N curves do not present any asymptote. So that, problems result in the assessment of the fatigue life. In these conditions, the concept of the fatigue limit has to be replaced by the fatigue strength at a fixed number of loading cycles. Temperature acquisitions during fatigue tests allow for a specific analysis that can support the researchers in understanding the complex processes that are involved in fatigue and their influence on fatigue life, even for aluminium alloys. In fact, the analysis of the surface temperature signal that was detected during a self-heating test provides a curve that is characterized by a distinct slope-change point at a specific stress value. Even though researchers have been investigating fatigue life characterisation and temperature variations for more than a decade, it is not clear what this point represents in terms of fatigue strength. The aim of the present paper is to find out a possible correlation between the thermal behaviour of AA5754-H111 undergoing self-heating testing procedure and fatigue strength at a specific loading cycles.

## 1. Introduction

The importance of the fatigue limit in mechanical design lies in the possibility to refer to a valid estimator that characterizes the indefinite life of a material or a component [1]. For certain materials, like structural steels, it is related to tensile strength [2]. This involves a rapid overview of the mechanical properties of the component.

The assessment of the fatigue limit derives from the assessment of S-N curve, the log-based relation between stress range, and the number of cycles [3], as the point of slope-change in the presence of a horizontal asymptote representing the infinite life. In case this “point” is not found, it means that the S-N curve tends to decrease continuously in the life regime fixed [4]. Because of this, it follows that even a little increment of stress may produce the failure of the material, and the concept of the fatigue limit has to be replaced by the fatigue resistance at fixed loading cycles. Moreover, several aluminium alloys are characterized by a specific behaviour exhibiting a double sloped trend between low cycle fatigue and high cycle fatigue, and between high cycle fatigue and very high cycle fatigue [5]. In this way, Mughrabi [6] found that the slope variations are principally due to the achievement of “crack initiation limit” at roughly around 10^6^ cycles, and the achievement of real fatigue limit in correspondence of which “cracks growth” at roughly 10^7^–10^8^ cycles, depending on grain dimensions as well as the specific microstructure [5]. However, the absence of “knee” point or the presence of different sloping trends in S-N curves [1,5,6,7,8,9,10,11] makes the assessment of fatigue life difficult, but the interest in this non-ferrous metal is growing due to the fact that it represents a good options for structural applications, particularly in the aerospace, aeronautic, naval, and automotive industries, due to their high strength to weight ratio and excellent resistance to atmospheric corrosion [12].

In these years, moreover, more and more researches have been focused on improving the performances of light alloys, like aluminium alloys, particularly the fatigue resistance by performing technological heat treatments, such as the age hardening [13]. In fact, the study of fatigue properties and fracture resistance is of critical importance specifically for commercial alloys, in order to reduce the time-to-market of the products. 

In this perspective, beside the traditional just exposed techniques to estimate the fatigue properties of the material, the techniques that are based on infrared thermography are becoming well established [14,15,16]. As it is well-known from literature, the standard methods, like S-N curve, do not allow for considerations about energy dissipated in the material, while thermographic techniques provide a more accurate approach since the temperature is strictly related to energy variations [17,18].

Some of the approaches that are based on thermography involve a reliable assessment of the fatigue heat sources capable of studying the damage of the material [19]. However, the applicability of such the technique to aluminium alloys is today questionable due to the high diffusivity properties that are involved in low temperature radiation detection. In effect, Krapez [20], by applying the Lock-in Thermography on AA7070, observed that the material temperature assessment was characterised by very low temperature variations even at high stress levels, and there was a non-distinct point separation in the temperature behaviour between non-damaged/damage conditions, in fact a slight variation of the slope of temperature increase was detected. This did not allow an easy identification of thermal and mechanical behaviour changes, as confirmed by other authors [21].

In the work of Li [22], the second order temperature variations “ΔTd”, due to non-isentropic energy dissipations, are presented for the riveted aluminium alloy 2A12. By analysing the thermograms the authors observed that the noise still prevailed on the thermographic signal, even at high loading levels. The same findings were presented by Morabito et al. [19] demonstrating a high signal-to-noise ratio in the thermal images of a fatigue loaded AA2024.

These issues justify the necessity of using accurate detectors, specific setup, and surface preparation.

The aim of the present paper is to correlate the thermal behaviour of the AA5754-H111 alloy that was tested by using a specific loading procedure [23], to the fatigue strength at a specific number of loading cycles. As it was just described, many authors [20,21,22,23] demonstrated as the fatigue limit of steels at 10^7^ cycles corresponds to the stress level that experiences the first significant heat dissipation in terms of temperature increase. So, this paper is the first approach to answer to the questions: “Does it exist a significant temperature variation in the thermal behaviour of aluminium?”, and by the way, “What does represent the significant temperature increase for materials such as aluminium alloys that do not present a horizontal asymptote in S-N curve?”. In this regard, a specific data assessment and a processing procedure will be presented capable of both determining estimation of the fatigue strength at a specific number of loading cycles and obtaining an “S-N curve” by thermal data.

To do this, a specific model of temperature was adopted for performing the analysis. The model allowed for the separation of the temperature components that is the separation between reversible temperature variations, which is also known as “thermoelastic” [24,25] temperature variations, and irreversible temperature variations that are associated with dissipative processes [26].

The thermoelastic component of temperature represents the reversible temperature variation promoted by the changes in elastic properties when the material is loaded under specific conditions [27]. 

In particular, the Thermoelastic Stress Analysis [24,25,27] provides a correlation between those temperature variations and surface stress field. Such the relationship is linear under an adiabatic condition. When the process is not adiabatic (heat diffusion, intrinsic damage sources), the relationship between temperature and stress becomes non-linear [28]. Whatever the relationship is, by studying thermoelastic temperature variations, one can study the fatigue behaviour and graphically detect the damage.

The irreversible temperature variations have been detected for the first time by [29] when he separated the elastic and plastic part of thermal behaviour of samples undergoing fatigue loading. These thermal changes are found occurring at twice the mechanical frequency, as they are related to the energy of intrinsic dissipations.

The adopted model for studying the temperature variations includes both of these components. In the present paper, the model has been applied to study the data obtained during the “self-heating” [23] loading procedure characterized by incremental applied load at the constant mechanical frequency of 17 Hz and the loading ratio R = 0.1. Such the method was already applied successfully on metals [30] and composites [31] in order to estimate the fatigue limit.

After the assessment of the thermal data, the rough thermal data were processed for obtaining the parameters (the different components of thermal signal) correlated to a specific aspect of the fatigue life of the material, which leads to study the fatigue strength of the material at a specific number of loading cycles. The novelty of the present paper is, also, represented by the possibility to use the data from the classic S-N curve in order to calibrate the temperature data in order to obtain a “thermographic S-N curve”.

## 2. Theory

The Thermoelastic Stress Analysis is a non-contact, non-destructive, full-field thermographic technique that provides the surface stress map, in terms of first invariant variations, by acquiring the surface temperature from a sample undergoing cyclic loading [27].

For homogenous, isentropic material, such the relation between temperature and stress is linear in case adiabatic condition are provided [25], otherwise it becomes non-linear.

The technique is widely used for performing non-destructive tests since the great potentiality is such that it is applicable to evaluate the surface state of stress of real components undergoing operating loading conditions [32]. In fact, another great advantage is that for performing thermoelastic measurement a simple preparation of sample is required; hence, it can be easily adopted in-situ.

The classical thermoelastic equation [24] provides reversible the temperature variations that are written as a function of the components running once and twice the mechanical exciting frequency f.

(1) $$\rho_{0}C_{\varepsilon }\frac{\delta T}{T_{0}}=-\left(\alpha -\frac{1}{E^{2}}\frac{\partial E}{\partial T}\sigma_{m}\right)\delta \sigma sin\omega t-\frac{1}{4E^{2}}\frac{\partial E}{\partial T}{\left(\delta \sigma \right)}^{2}cos2\omega t,$$

Equation (1) is written for the one-dimensional stress state.

In Equation (1), the $\rho_{0}$ is the density of the unstrained material, $C_{\varepsilon }$ is the specific heat under constant strain, $T_{0}$ is the reference temperature, *E* is the Young’s modulus, $\sigma_{m}$ is the applied mean stress, δσ is the stress amplitude, and ω the pulsation of the system proportional to 2πf.

From Equation (1), it follows that for material where its properties are temperature dependent, the first component of temperature variations depends on both mean stress and stress amplitude, while the second order component amplitude determines a square of stress amplitude dependence of the temperature variations, which ever exists. Generally, the term ($\frac{1}{4E^{2}}\frac{\partial E}{\partial T}$) is expected to be small, but its influence increases as the stress amplitude increases [27].

In this case, the material mechanical properties do not depend on the temperature, the Equation (1) transforms in:

(2) $$\rho_{0}C_{\varepsilon }\frac{\delta T}{T_{0}}=-\alpha \delta \sigma sin\omega t,$$

**Figure 1:**
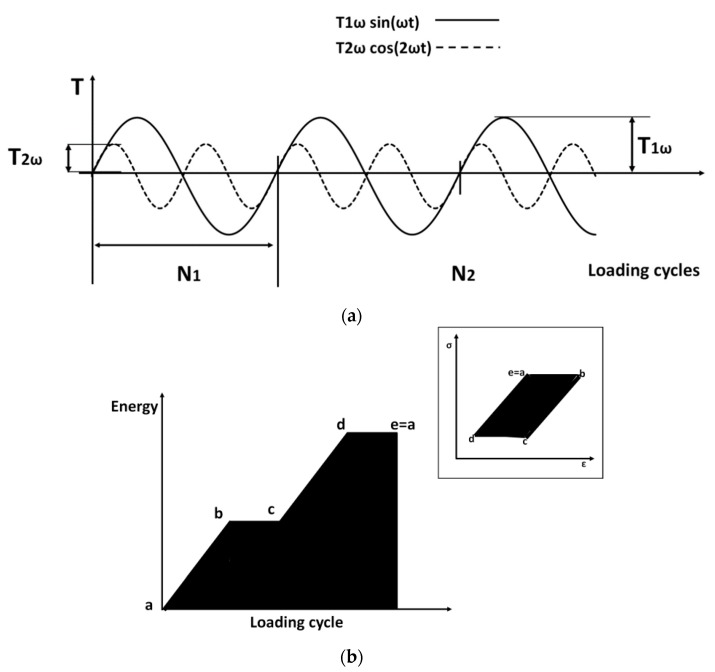
Thermoelastic components (**a**) energy increase from hysteresis loop (**b**).

Figure 1a (amplitude indicated as $T_{1\omega }$) reports all of the temperature variations for a generic material that was tested at a generic loading condition. Besides the first order temperature variations, as just said, the thermoelastic stress analysis provides also a second order temperature variation that was running at twice the mechanical frequency (indicated as $T_{2\omega })$, as also reported in Figure 1a.

Beyond the reversible temperature contributions, there are the temperature contribution produced by the irreversible energy production in the material due to the presence of a hysteresis loop that is produced by the strain retardation with respect to the imposed stress [33].

The reversible energy variations clearly produce a zero mean temperature increase during a loading cycle. However, they are instantaneously different from zero, as represented in Figure 1a. When damage processes occur, the reversible temperature variations sum up with those that are related to dissipative phenomena [29,31,34].

The dissipative phenomena are related to the presence of a hysteretic behaviour. In this condition, the strain increases two times per cycle, and such the phenomenon generates an increase in the intrinsic energy, in turn, producing the temperature increase. As previously said, the second order temperature increase due to irreversible phenomena that are related to the damage, is summed up to the second order reversible temperature variations, making their separation quite difficult.

In Figure 1b, it is possible to observe the energy from hysteresis loop (area under generic bilinear hysteresis loop) and the energy producing the temperature irreversible increase (area under the temperature increase), as modelled in [35].

All the contributions to the temperature of the material can be assessed by using a specific model, which will be discussed in further paragraph. Further paragraphs, will also show the accurate filtering procedures for taking into account reversible and irreversible temperature variations in order to assess an estimation of the fatigue strength of the material.

## 3. Material and Setup

The tested material is the aluminium alloy 5754-H111, which is used for some components in the aerospace and automotive fields, Table 1.

**Figure 2:**
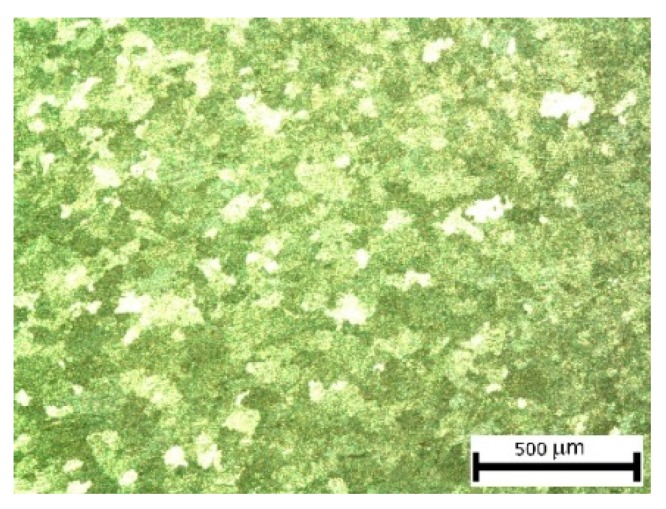
Grain dispersion of AA5754-H111 at light microscopy.

The thermo-mechanical properties of this material have been extensively studied in the work of De Filippis et al. [36]. In Figure 2, is reported the micrograph of two areas of a sample. After thermal treatment, the microstructure presents large grains so that the expected behaviour is ductile represented by large strains.

**Figure 3:**
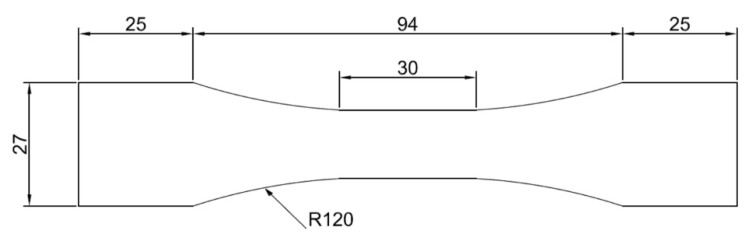
Sample geometry and dimensions expressed in mm [37].

The samples have been designed according the Standard ISO 6892-1:2016 [37] and the geometry is typically “dog-bone”, as represented in Figure 3.

The fatigue tests, called self-heating [38,39], provide a gradual increase of the stress up to the sample failure. Table 2 reports the imposed stress levels (denominated as “step”), the underlined values correspond to the same stress values that are imposed to the sample during S-N experimental campaign. For each stress level, the stress amplitude (Δ*σ*), the maximum stress ($\sigma_{max})$ have been reported. The mechanical properties have been determined by performing tensile tests [37] on five samples. The test was performed in stress (load) control, so that, according to the standard, the loading rate chosen was 5 MPa/s. The results in terms of Ultimate tensile strength and Young modulus are, respectively, 210.70 MPa (standard deviation 3.11 MPa on five tests) and 70.00 GPa (standard deviation of 486.01 MPa on five tests).

**Figure 4:**
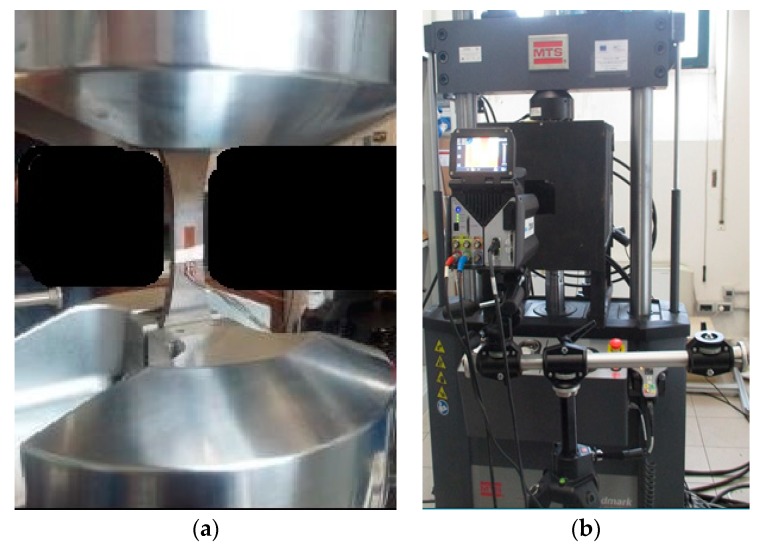
Equipment and setup: (**a**) strain gage applied on sample surface; (**b**) IR detector monitoring the test.

The tensile properties of the materials are useful for determining the initial stress of self-heating, that in this case, is roughly 20% of the ultimate tensile strength of the material. The tension-tension cyclic tests run at 17 Hz at fixed stress ratio of 0.1, for 20,000 cycles of loading machine (MTS_370 capacity 100 kN). After the 20,000 cycles, the stress increase was achieved. During each loading block, an infrared IR camera acquired thermal radiation of one face of the sample gage length (Figure 4).

The adopted detector was the cooled Flir X6540sc (pixel matrix 640 × 512 and NETD <25 mK).

The detector acquired three sequences every 6000 cycles of loading machine at 100 Hz for ten seconds. The results that are presented in this paper refer to the mean value between the three data acquisitions.

**Figure 5:**
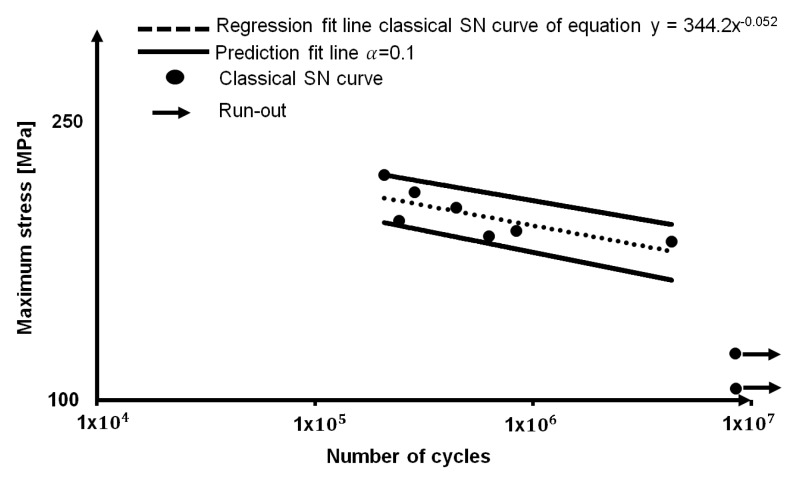
S-N curve and prediction interval for AA 5754-H111.

An S-N experimental curve has been performed on nine samples, in order to obtain an estimation of the fatigue strength of the material. The loading table is reported Table 3. The run-out considered, as an index of fatigue life is 10^7^, as suggested by the Eurocode EN 1999-1-3. The result in terms of the fatigue life at 10^7^ was 148.30 MPa, the prediction fitting line was calculated by using *α* = 0.1, as reported in Figure 5.

## 4. Data Processing

The processing of the data is an important step of the analysis as it provides the different temperature components. The model is represented by the Equation (3) and it is based on previous studies reported in [20,30]:

(3) $$S\left(t\right)=S_{0}+bt+S_{1}\sin\left(\omega t+\varphi_{1}\right)+S_{2}\cos\left(2\omega t+\varphi_{d}\right)$$

*S*(*t*) is the thermal signal at fixed time instant of the single pixel of the focal plane array which can be decomposed in its contributions: $S_{0}$ is the signal offset that is representing the influence of environment on temperature of the body, *bt* the component that represents the linear increase of the temperature signal, $S_{1}\sin\left(\omega t+\varphi_{1}\right)$ the first harmonic component that is characterized by the amplitude signal $S_{1}$, which represents the amplitude of the thermoelastic temperature signal variations (previously indicated as $T_{1\omega })$, and the $\varphi_{1}$ phase shift, the $S_{2}\cos\left(2\omega t+\varphi_{d}\right)$ the second harmonic component that amplitude $S_{2}$ includes both the reversible temperature signal variations that are related to thermoelastic effect (previously indicated as $T_{2\omega })$ and irreversible temperature variations related to intrinsic dissipations. These latter components running at twice the mechanical frequency (2ωt) are shifted by the angle $\varphi_{d}$.

**Figure 6:**
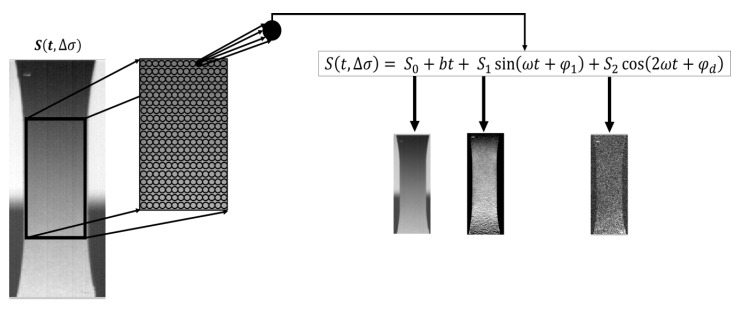
Processing procedure: temperature signal variation outputs.

The Equation (3) is implemented in the software IRTA^®^ that provides not only quantitative values for each parameter, but also the related maps, which are the matrixes of pixels whose values are specific for each thermal component. The overall procedure, for the generic samples that is loaded at specific Δ*σ* is represented in Figure 6. Further, Figure 6 depicts the three maps of signal that is related to the temperature signal components of interest. An important observation that is possible to make by observing Figure 6, is such that each map provides different information that contributes to understand the behaviour of the material during fatigue loadings. Specifically, both *S*_1_ and *S*_2_ highlight very localized phenomena in the gage length, while *S*_0_ provides an overall map of the temperature in the gage length.

**Figure 7:**
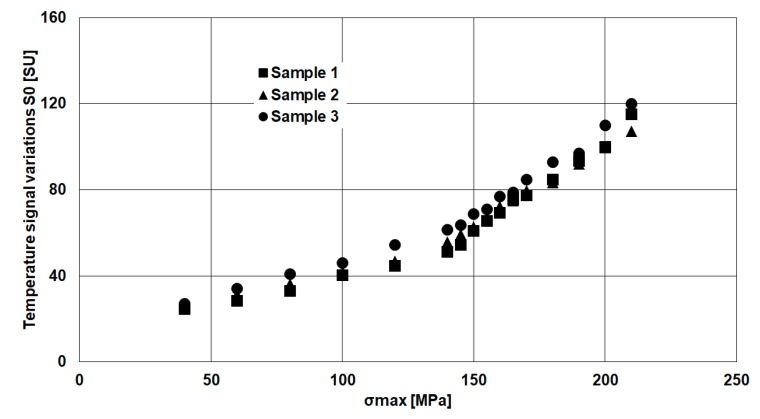
Temperature signal variations (*S*_0_): rough data.

Figure 7 quantitatively depicts the *S*_0_ parameter, which represents the total temperature variation.

The *S*_0_ signal presents a double-sloped trend due to different disturbing heat sources, such as the heating from convection and conduction. The first contribute is represented by the temperature of environment, while the second one can be determined by the heating from moving grip of loading machine that is in contact with hot oil. The influence of these disturbing heat sources was discussed in [23], however this particular temperature signal behaviour can be totally filtered out by using the procedure that was adopted for estimating fatigue strength, as will be explained in further paragraph.

**Figure 8:**
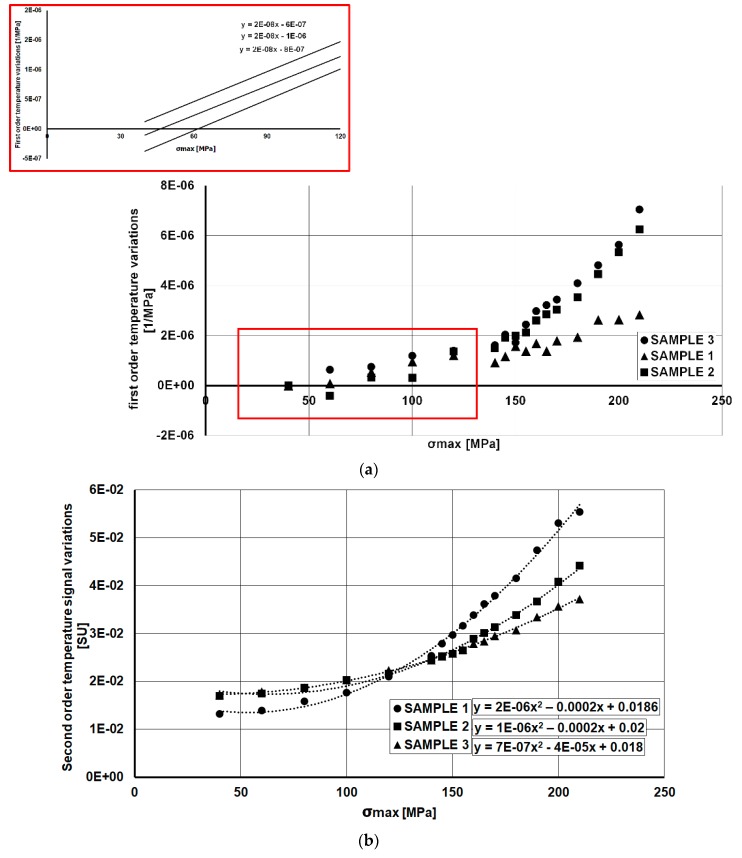
Rough data of the thermal signal components: *S*_1_ (**a**) *S*_2_ (**b**).

Figure 8a reports the *S*_1_ parameter data. As shown in the theory, this component of the temperature is related to the thermoelastic first order effect that is influenced by the mean stress and the stress amplitude.

In effect, the signal that is reported in Figure 8a has been normalized by stress amplitude Δ*σ*. It means that each values of temperature in gage length was divided for the specific stress level (Table 2). However, the data are reported in respect of maximum stress ($\sigma_{max}$) that is related to stress amplitude Δ*σ* and stress ratio *R*, by Equation (4):

(4) $$\Delta \sigma =\sigma_{max}\;\left(1-R\right)$$

The thermoelastic effect due to the mean stress is clear for this aluminium alloy. Such dependence is reproducible through the samples, in fact, as depicted in Figure 8a, the slope of the first points lying in elastic loading regime are sloped of the same quantity.

The second order temperature variations that are reported in Figure 8b exhibit a Δ*σ*^2^ dependence, as shown by Equation (1), so that the curve fitting is represented by a polynomial second order curve. Particularly for the *S*_2_ parameter, the study of the fatigue behaviour can be difficult since it includes both dissipative and thermoelastic effects, in other words, it includes both reversible and irreversible temperature variations. So, to discern these two effects, a filtering procedure is needed as will be explained in further paragraphs.

In further paragraphs, it will be presented the results in quantitatively terms and graphically. The comparison with the S-N curve will also be presented in order to understand the relationship between the signal (temperature) variations measured and the experimental S-N curve.

## 5. Results and Discussion

### 5.1. Assessment of Significant Thermal Signal Variations by Using the Threshold Method

In order to assess a significant value for the signal variations *S*_1_, *S*_2_, *S*_0_, the data were addressed to a specific procedure, as applied on ferrous metals [23] as well as on composites [31].

The procedure that is presented in [23] is based on evaluating the fatigue strength of the material by analysing the temperature signal variations. In the reported work of De Finis [23], the method was applied just to *S*_0_ data. In the present work, an extension of the procedure to the signals *S*_1_ and *S*_2_ is presented.

In particular, the procedure deals with the assessment of the point at which the specific temperature signal variation is significant with respect to the data series representing an “undamaged condition”. This latter is characterized by zero temperature variations, while the significant parameter variation that is found is considered as an index of fatigue resistance. 

The procedure is based on evaluating the threshold value of residual data. In view of this, an initial regression of the first data couples ($\sigma_{max},\;S_{x})$ with *x* = 0, 1, 2, is made. The regression is linear for *S*_1_ and *S*_0_, while it is polynomial (the grade of interpolating polynomial is 2) for *S*_2_. The difference between two methods (linear and polynomial) is due the square stress amplitude dependence of the second order temperature variations parameter with respect *S*_1_. However, this procedure allows for processing the data in order to eliminate both thermoelastic influences on *S*_1_, *S*_2_, and the influence of “disturbing heat sources” on *S*_0_.

**Figure 9:**
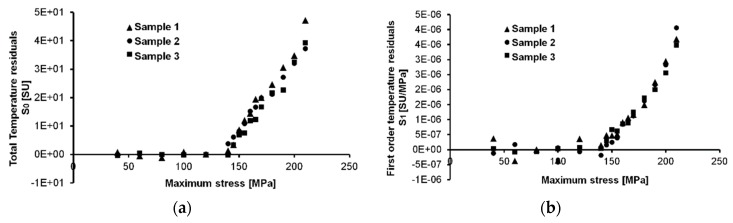

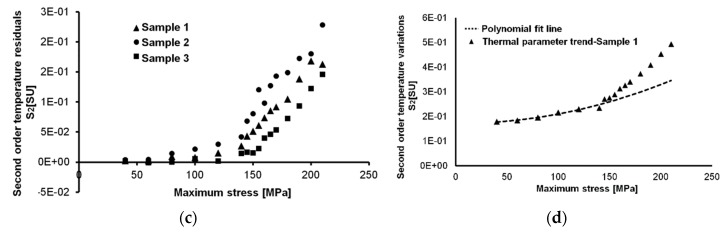
Linear Residuals of *S*_0_ (**a**) and *S*_1_ (**b**) parameters, and polynomial residuals of *S*_2_ parameter (**c**), example of the polynomial fit line adopted for analysing sample 1 (**d**).

The outputs of the aforementioned procedure are reported in Figure 9a–c. In Figure 9a, the linear regression of the initial data couples ($\sigma_{max},\;S_{0})$ allows for filtering out the thermal contributions that are represented by the heat exchanged by convection and conduction. In Figure 9b, apart from a little data scatter at initial loading levels due to (high noise to signal ratio), the mean stress effect is eliminated from first order thermal signal variations.

For $S_{0}$ and $S_{1}$, the same analysis procedure of the data can also allow for the data processing providing a simplification and a time reduction of the processing steps.

The polynomial data regression algorithm to which the second order temperature variations $S_{2}$ was added, produce a quasi-zero temperature signal variation at lower imposed stresses, and a more pronounced scatter at higher stresses. This could be due to the fact that the $S_{2}$ are related to the energy of intrinsic dissipations that are different from sample to sample, since it could be different from the damage processes. Otherwise, the issue could be related to the number of the points that are considered in polynomial interpolation. In particular, in Figure 9d, the polynomial interpolation has been made on five initial data couples ($\sigma_{max},\;S_{2})$. For the purpose of assessing the fatigue strength of the material, the threshold method provides the first point (temperature signal data) at which a regime transition in the behaviour of the material occurs. This behaviour variation can be represented by the statistical significance of the temperature signal increase with respect to the data series representing the “undamaged” conditions. 

The procedure for estimating the fatigue strength by using a threshold is well-known, so that the reader is invited to refer to the paper [23] for an in-depth explanation of the different steps for the assessment of the threshold *µ* + 6*σ*.

The results of the application of the method on *S*_0_, *S*_1_, and *S*_2_ are reported in Table 4.

The fatigue strength that is found by using *S*_0_ and *S*_2_ parameters is slightly the same (approximately 145 MPa), while the results that are provided by the application of the method on the S_1_ parameter indicate a higher value with a higher scatter of data. 

In order to make sense of these values it is interesting to correlate these stress values that were obtained with those that were reported in the S-N curve. In this way, a correlation between thermal signal and fatigue strength of the material is assessed. 

The total number of samples that were tested for assessing the S-N curve in a classical way is limited. In this regard, the thermography can support exploring the overall fatigue life of the material without performing further tests. In particular, by simply using the *S*_2_ data, it is possible to obtain a “thermographic S-N curve” that allows for reducing the testing time and the costs of the experimental campaign, as will be detailed explained in the further paragraph.

### 5.2. Correlation between S_2_ Thermal Signal Variations and Experimental Data to Obtain “Thermal S-N Curve”

In this section, the correlation between thermal data and those that were provided by experimental S-N curve is reported in order to reduce both the testing time and the costs of the experimental campaign. 

The procedure starts by considering the parameter that is related to intrinsic energy dissipations that are occurring in the presence of irreversible phenomena in the material, such a parameter is represented by the second order temperature variations $S_{2}$.

The residuals of $S_{2}$ are considered for the analysis since the evaluation of the residuals eliminates completely the thermoelastic reversible second order effect.

The residuals values of $S_{2}$ considered are those in correspondence of the stress values that are imposed during the S-N experimental campaign that is reported in Table 3, specifically they refer to the underlined values that are reported in Table 2.

**Figure 10:**
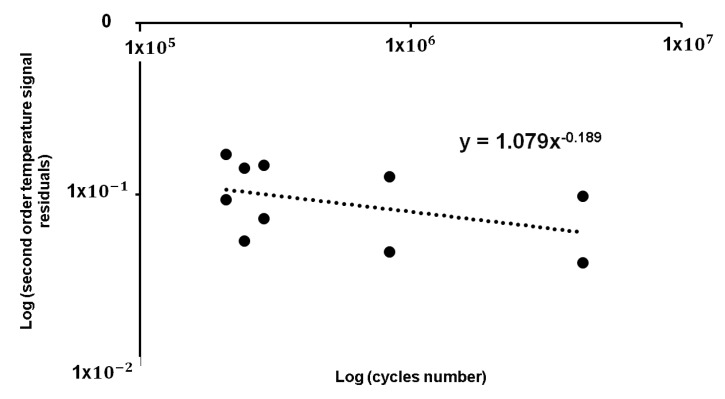
Polynomial residuals of S_2_ parameter in relation to the number of cycles of a classical S-N curve.

A model was built based on the $S_{2}$ residual values of the samples two and three, as represented in the graph of Figure 10 in relation to the cycles number of the classical S-N curve that is reported in Table 3. The axes in Figure 10 report logarithmic values.

The equation of the model can be expressed in the form of Equation (5):

(5) $$S_{2}=aN^{b}$$

where $S_{2}$ represents the residuals values of second order temperature variations, *N* corresponds to the cycles number that was obtained by performing the S-N curve, the values are reported in Table 3, and the coefficients *a* and *b*, are experimentally assessed.

By using the coefficients of Equation (5) and the values of residuals $S_{2}$ of the sample one, it is possible to assess the number of cycles of the calibrated thermal S-N curve. Table 5 reports the results of the just discussed calculations for the sample one.

**Figure 11:**
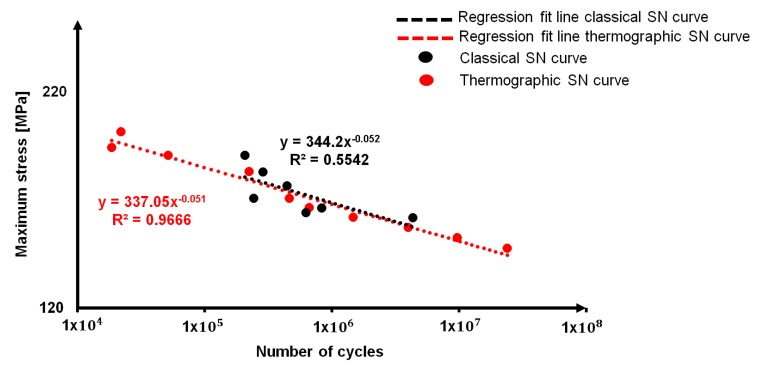
Classical S-N curve and thermographic S-N curve.

By plotting for sample one, the loading cycles of Table 5 as function of stress levels of classical S-N curve, it is possible to obtain the “thermographic S-N curve”, which is reported in Figure 11 correlated to the classical S-N curve in double logarithmic graph.

As it is possible to observe in Table 5, the model was applied to a wider range of stress level in comparison with those that are reported in Table 3, in order to predict the fatigue life of the material under different aspects, ranging from low-cycle fatigue to high-cycle fatigue. The fatigue life at the runout that is indicated by the Eurocode [40], 10^7^ cycles, is indicated.

In this way, by using the points of classical fatigue curve for the material and just three specimens that were obtained by performing three self-heating tests, a complete screening of the fatigue life of AA5754-H111 is assessed.

This approach allows for a reduction in the time and costs of the experimental campaign [41].

In next section, the stresses that were obtained by applying threshold method on *S*_1_, *S*_2_, and *S*_0_, will be correlated to the thermographic S-N curve in order to better understand what the value that was obtained by applying the threshold value corresponds to.

### 5.3. Estimation of the Fatigue Life at Specific Loading Cycles by Using the “Thermal S-N Curves”

Despite that aluminium alloys do not present there a horizontal asymptote in S-N curve (fatigue limit) [1], it is however possible to evaluate the fatigue strength at a specific number of loading cycles by using the thermographic approach. 

**Figure 12:**
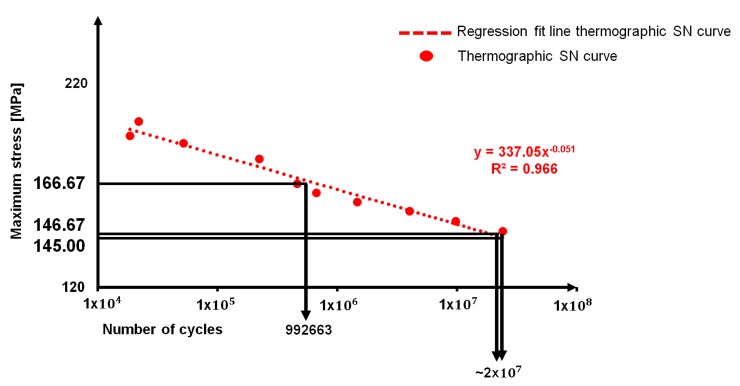
Assessment of the fatigue strength of AA5754-H111 by using the data from threshold method and thermographic S-N curve.

Table 6 reports the values obtained by performing the threshold method on *S*_0_, *S*_1_, and *S*_2_ thermographic data and in Figure 12 the correlation between thermographic S-N curve data and the stresses resulting from the application of the threshold method are shown.

By relating the data presented in Table 6 with the S-N curve, as reported in Figure 12, it is possible to observe that the fatigue strength estimated with *S*_2_ and *S*_0_ (respectively, 146.67 MPa and 145 MPa) is higher (2 × 10^7^) than the one in correspondence of the runout 10^7^ cycles (148.30 MPa).

The fatigue strength that was obtained by *S*_1_ data provides a consistently higher value of stress in correspondence of a fatigue life of 9.69 × 10^5^ cycles.

Even if the results of *S*_0_ and *S*_2_ appear to be different from the one that is provided by *S*_1_, it is worth noting that it can be related to different aspect of the fatigue life of material. In fact, together with the absence of horizontal asymptote in the High Cycle Fatigue (HCF) regime, as reported in Figure 5 and Figure 11, accordingly with the literature [5,6,7,8,9], the S-N curve presents a double sloped trend at approximately 10^6^ cycles. Even if in literature, on the presented alloy, there are few information on fatigue behaviour, several authors [1,2,3,4,5] discussed of the double slope variation of the curve in sight of the absence of a fatigue limit, especially in the High Cycle Fatigue (HCF) regime, where the fatigue limit of several type of steels lies. In particular, on alloys 7075-T6 and 2024-T3, Newman [8] observed a slope variation at approximately 8 × 10^5^ loading cycles.

**Figure 13:**
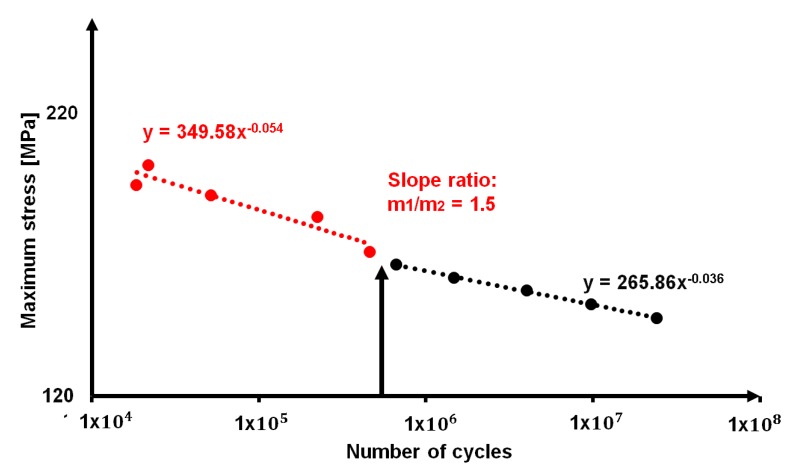
Persistent slip band limit provided by the results of *S*_1_ parameters.

By splitting the thermographic SN curve in two separated data series, Figure 13, it is possible to detect that there is a change in the slope approximately at 10^6^ cycles. This change in slope, as stated by [6], corresponds to the achievement of persistent slip band limit that determines the increase in deformation and in turn the onset of damage phenomena. The persistent slip band limit separates the Low Cycle Fatigue (LCF) where the stress values are correlated to the necessary energy to move dislocations and creating the persistent slip bands structures [5].

In this regard, *S*_1_ seems to be a good estimator of the stress condition at which the regime switches from LCF to HCF, since the fatigue life seems to be very similar to the point of inversion of the S-N trend that is found for present alloy at 7 × 10^5^ cycles.

On the other hand, the parameters *S*_0_, *S*_2_ seem to be providing an estimation of the fatigue life in very high cycle regime at higher values than 10^7^ cycles.

Another goal of the present analysis is represented by the possibility that the thermographic S-N curve can reproduce all the aspects of the fatigue behaviour of the material, and it somehow can be used for separating the behaviour of material when the crack initiates and the one that is characterised by the growth of the cracks. Hence, from macroscopic measurements, one can achieve microscopic evaluation related to microstructural processes.

## 6. Conclusions

In this paper, a thermographic approach has been proposed capable of relating the thermal behaviour of AA5754-H111 to the fatigue life estimations based on S-N curve. The results of the application of a specific loading procedure “self-heating” have been presented and correlated to the standard reference, and furthermore, a thermographic S-N curve has been proposed.

The aluminium is a problematic nonferrous metal due to both its high diffusive behaviour together with the “duplex nature” of S-N curve that reports different slope changes and several issues results from the understanding of the fatigue behaviour.

These behaviours make both thermal characterization as well as the mechanical characterization difficult.

Even if the presence of these issues, a procedure for filtering data and obtaining the thermal parameters *S*_0_ has been presented that can eliminate the disturbing heat contributions due to influence of environment or conduction throughout the material. The procedure also allows for filtering out thermoelastic mean stress or square stress amplitude dependences from the thermal component related to first order *S*_1_ and second order *S*_2_ temperature variations.

Moreover, since the aluminium does not present a horizontal asymptote in the S-N curve, it was correlated the stress that was found by using each of parameters provided by the thermals signal analysis and it was assessed the estimation of the fatigue limit at fixed number of cycles. In particular, the *S*_0_ and *S*_2_ estimate a stress value over the conventional run-out of 10^7^ cycles, while the *S*_1_ provides values that are similar to the first slope variation, which corresponds to the transition between LCF and HCF limit in correspondence of the critical stress to which a generic damage process growth.

Furthermore, the data from classical S-N curve have been used for calibrating thermographic data, in terms of second order component *S*_2_, in order to obtain a thermographic S-N curve that allows for exploring the different aspects of the fatigue life of the materials with less time and less cost in comparison to the traditional experimental campaign.

The proposed correlation between estimation of fatigue life by thermal data and S-N curve provided encouraging results for the development of a rapid way to study the fatigue behaviour of aluminium alloys.

## Figures and Tables

**Table 1:** Chemical composition of the 5754-H111 aluminium alloy.

Alloy	Si	Fe	Cu	Mn	Mg	Cr	Ni	Zn	Ti	Other Elements
AA5754-H111	0.40	0.40	0.10	0.50	2.60–3.60	0.30	0.05	0.20	0.15	0.05

**Table 2:** Loading levels for self-heating tests.

Loading Levels					
Step	Δ*σ*	*σ_max_*	Step	Δ*σ*	*σ_max_*
	(MPa)	(MPa)		(MPa)	(MPa)
1	36.0	40.0	8	135.0	150.0
2	54.0	60.0	9	139.5	155.0
3	72.0	80.0	10	144.0	160.0
4	90.0	100.0	11	148.5	165.0
5	108.0	120.0	12	153.0	170.0
6	126.0	140.0	13	166.5	185.0
7	130.5	145.0	14	175.5	195.0

**Table 3:** S-N curve loading levels applied.

Sample	*σ_max_* (MPa)	Number of Cycles	Sample	*σ_max_* (MPa)	Number of Cycles
1	70.0	1 × 10^7^	5	170.0	242,637
2	100.0	1 × 10^7^	6	185.0	286,216
3	177.0	444,272	7	165.0	835,571
4	160.0	4,323,679	8	162.5	626,212
-	-	-	9	195.0	207,560

**Table 4:** Application of the threshold method on thermal signals.

Samples	Fatigue Strength Assessment: Threshold Method Applied on Different Thermal Indexes (Values in MPa)			
	S_0_	S_1_	S_2_	CLASSICAL S-N CURVE
SAMPLE 1	150.00	190.00	140.00	-
SAMPLE 2	140.00	160.00	145.00	
SAMPLE 3	145.00	150.00	155.00	
Mean (MPa)	145.00	166.67	146.67	Mean (MPa) 148.30
Std. Dev (MPa)	5.00	20.82	7.64	Prediction Interval. 90% (MPa) 11.89

**Table 5:** Evaluation of number of cycles of thermographic S-N curve by using the model of Equation (5).

Residuals of $S_{2}$ of Sample 1 (Signal Units)	$\sigma_{max}$ (MPa)	Number of Cycles
0.04	145	2.41 × 10^7^
0.05	150	9.77 × 10^6^
0.06	155	4.01 × 10^6^
0.07	160	1.47 × 10^6^
0.09	165	6.67 × 10^5^
0.09	170	4.63 × 10^5^
0.11	185	2.24 × 10^5^
0.14	195	5.19 × 10^4^

**Table 6:** Fatigue life estimations by using the stress provided by the threshold method.

Parameter	Fatigue Strength Provided by Threshold Method (MPa)	Fatigue Life Estimated (Number of Cycles)	Fatigue Life Estimated at 10^7^ by Classical SN Curve (MPa)
*S*_0_	145.00	>10^7^	148.30
*S*_1_	166.67	<10^7^; ~10^6^	
*S*_2_	146.67	>10^7^	
